# Mapping Men’s Mental Health Help-Seeking After an Intimate Partner Relationship Break-Up

**DOI:** 10.1177/10497323221110974

**Published:** 2022-06-25

**Authors:** John L. Oliffe, Mary T. Kelly, Gabriela Gonzalez Montaner, Zac E. Seidler, David Kealy, John S. Ogrodniczuk, Simon M. Rice

**Affiliations:** 1 School of Nursing, 8166 University of British Columbia, Vancouver, BC, Canada; 2 Department of Nursing, 110600 The University of Melbourne, Carlton, VIC, Australia; 3 188668 Orygen, Parkville, VIC, Australia; 4 Centre for Youth Mental Health, 590696 The University of Melbourne, Melbourne, VIC, Australia; 5 376015 Movember Foundation, East Melbourne, VIC, Australia; 6 Department of Psychiatry, 175098 The University of British Columbia, Vancouver, BC, Canada

**Keywords:** men’s mental health help-seeking, masculine self-reliance, men’s intimate partner relationship break-ups, family dissolution, masculinity

## Abstract

Deleterious effects of separation and divorce on men’s mental health are well-documented; however, little is known about their help-seeking when adjusting to these all-too-common life transitions. Employing interpretive descriptive methods, interviews with 47 men exploring their mental health help-seeking after a relationship break-up were analyzed in deriving three themes: (1) Solitary work and tapping established connections, (2) Reaching out to make new connections, and (3) Engaging professional mental health care. Men relying on solitary work and established connections accessed relationship-focused self-help books, online resources, and confided in friends and/or family. Some participants supplemented solitary work by reaching out to make new connections including peer-based men’s groups and education and social activities. Comprising first-time, returning, and continuing users, many men responded to relationship break-up crises by engaging professional mental health care. The findings challenge longstanding commentaries that men actively avoid mental health promotion by illuminating wide-ranging help resources.

## Introduction

The breakdown of an intimate partner relationship can trigger and/or exacerbate men’s mental illness, increasing their risk for anxiety, depression, and suicide (Oliffe et al., 2022). Though such mental health challenges can prompt men’s help-seeking, there is much debate about the role of masculinity in men’s use of professional mental health care services (Seidler et al., 2018). In gendered contexts, masculine norms and ideals—most prominently self-reliance—have featured as a pivot that can constrain (Addis & Mahalik, 2003; Mahalik & Di Bianca, 2021; Pirkis et al., 2017) or enable (Struszczyk et al., 2019) men’s engagement with professional mental health care. Herein, the predominance of professional mental health care services as what legitimately counts as *the help* has also limited depictions of, and insights to men’s mental health help-seeking (Fletcher & St George, 2010; Harding & Fox, 2015). The current study maps men’s mental health help-seeking after an intimate partner relationship break-up, highlighting the gendered dimensions for what participants access and experience as mental health help.

### Men’s Mental Health and Intimate Relationship Breakdowns

Many men experience the breakdown of an intimate partner relationship as one of life’s most challenging events (Stack & Scourfield, 2015) wherein factors including social isolation, estrangement from children, and the division of assets can entwine to increase their risk for mental illness and suicide (Evans et al., 2016; Shiner et al., 2009; Stack & Scourfield, 2015). Sex differences research consistently reports that males have greater risk for suicidality (suicidal ideation, behaviors, and/or attempts) after a relationship break-up (Evans et al., 2016), with divorced men being 8-times more likely to die by suicide compared to divorced women (Kposowa, 2003). Although men may conceal their distress publicly, they are more likely than women to anonymously seek-help for their relationships online and/or express their emotions (i.e., heartache) (Entwistle et al., 2021). Masculinities theory (Connell, 2005) can provide important insights to the gendered dimensions of these (and other) sex differences pertaining to intimate partner relationships. For example, partner-initiated break-ups are especially injurious but many men attempt to downplay their hurt and re-establish their manhood by quickly and unemotionally moving on to another romantic relationship (Hartman, 2017). Although life stage and relationship specificities can also impact distress levels, men who conceal what is felt and/or self-medicate with substances (Turner et al., 2018) have increased risk for male depressive symptoms including anger and risk-taking (Rice et al., 2020). Men who internalize their emotions relating to relationship break-up (i.e., guilt and shame) have increased risk for self-directed anger, harm, and suicide (Shiner et al., 2009) due to a loss of masculine status (Scourfield & Evans, 2015). In addition, there are claims that thwarted control and partner betrayal, and the loss of custody of children in relationship break-ups can provoke male suicide as punishment for ex-partners leaving (River & Flood, 2021). Connections between masculinities, mental illness and suicide, and the dissolution of intimate partner relationships have drawn research attention (Evans et al., 2016; River & Flood, 2021; Scourfield & Evans, 2015; Shiner et al., 2009), however, very little is known about men’s mental health help-seeking in the aftermath of a relationship break-up. This lack of research progress has stymied intervention advancement regarding the types of services and supports (prior to, and post relationship dissolution) that should be offered to men to enhance the effectiveness of their relational skills.

### Masculinity and Men’s Mental Health Help-Seeking

The most often told story of men’s mental health help-seeking belabors conformity to specific masculine norms to explain (and predict) men’s estrangement from professional care services. For example, Addis and Mahalik (2003) report masculine self-reliance and emotional control as underpinning men’s reticence for accessing and resistance to engaging professional mental health care. Self-reliance and suicidal thoughts have also been coupled to explain why men are unlikely to talk through problems or seek help from friends and family when feeling down or vulnerable (Pirkis et al., 2017). When depression threatens men, Mahalik and Di Bianca (2021) further suggest self-reliance is levered by men to re-establish their manhood. Qualitative work has consistently challenged these deficit depictions of self-reliance, contextualizing how a plurality of masculinities can be embodied to work for men’s mental health help-seeking (Emslie et al., 2006; Ferlatte et al., 2019; Oliffe et al., 2021; Player et al., 2015). Herein, men’s talk (Emslie et al., 2006), strength-based recovery efforts (Oliffe et al., 2021), resilience (Ferlatte et al., 2019), and self-health to protect significant others (Player et al., 2015) can norm mental health help-seeking. There has also been research attention to the provision and gendered fit of mental health services to more fully contextualize men’s help-seeking experiences. Lack of connection with the therapist, slow progress in therapy (Seidler et al., 2021), misguided assumptions, and outdated ideas for what mental health consults entail (Harding & Fox, 2015) preside as factors underpinning men’s dropout from professional mental health care services. Inversely, forging engagement with services are men’s beliefs about the benefits of professional care and familiarity with the mental health care system (Seidler et al., 2021).

There have also been calls to broaden what counts as help beyond the predominance of professional mental health care services as a means to fully account for (and cater to) men’s help-seeking (Fletcher & St George, 2010). Herein, men’s use of relationship self-help books (Doss et al., 2009) and online mental health resources and forums (Entwistle et al., 2021; Oliffe et al., 2020) emerge as somewhat normative, and perhaps mainstream contemporary masculine practices. Likewise, informal supports including family, friends, and community-based programs meet the mental health needs of many men. For example, fathers’ resourceful help-seeking when separating from their partner and child [ren] highlighted the mental health benefits of engaging information and emotional help from family and friends (Fletcher & St George, 2010). That men are more amenable to help-seeking if they have the opportunity to reciprocate (Addis & Mahalik, 2003) may also go some way to explaining the rise of men’s community-based mental health programs (Oliffe et al., 2020). Indeed, help-seeking normativity perceptions are influenced by other men’s health behaviors, though the influence of such role-modeling for bridging to professional mental health care services are poorly understood (Mahalik et al, 2007). Chandler (2021) also cautions that dominant masculine discourses can bar men from peer as well as professional mental health care.

By mapping men’s mental health help-seeking after an intimate partner relationship break-up, the current study highlights the gendered dimensions for wide-ranging help options, much of which reveals strong potential for advancing men’s wellbeing.

## Methods

Guided by interpretive description (ID) methodologies (Thorne, 2016), the focus was on adapting virtual methods comprising individual Zoom interviews to facilitate discussions with men about their help-seeking in the context of experiencing an intimate relationship break-up (Oliffe et al., 2021). In line with ID, constant comparative analytics were used to analyze the data and transition the inductively derived findings to detail practice-based recommendations.

### Data Collection

With University ethics approval, Australian and Canadian based men who were at least 18-years-old and had experienced the break-up of an intimate partner relationship were recruited via online advertisements (Twitter, Reddit, and Facebook). The study recruitment flyers indicated our interest in interviewing men who had experienced a separation, divorce, or relationship break-up as a means to explore their mental health perspectives and practices. Men were screened for eligibility by the project manager, and following consent and demographics an individual semi-structured interview was scheduled for a mutually convenient time. Using an interview guide, in-depth discussions were facilitated by four trained researchers (two female and two male) based in Canada. The digitally recorded interviews were completed in 2020 and lasted 60–90 min, and participants received a $100 e-gift card to acknowledge their time and contribution to the study. The interviews included an emphasis on participants’ mental health help-seeking to understand what men valued as help generally, as well as specifically for their disrupted intimate partner relationships (i.e., divorce, separation, and break-up). Interview questions included, “a*fter the relationship ended what were your thoughts about and strategies for moving on?*” *and* “w*hat were the best forms of help that you accessed in the aftermath of the relationship ending?*” The digitally recorded interviews were downloaded, transcribed verbatim, accuracy checked, and cleaned to remove identifying participant details.

### Sample

Forty-seven men residing in Canada (*n* = 29; 61.7%) and Australia (*n* = 18; 38.3%), ranging 26 to 70-year-old (M = 40.87 years; SD = 10.59 years), took part in the study. Participants self-identified as heterosexual (*n* = 38; 80.9%), gay (*n* = 7; 14.9%) and bisexual (*n* = 2; 4.3%), and most were separated or divorced (*n* = 26; 55.3%) and currently single (*n* = 33; 70%). Relationship duration ranged from 4 months to 28 years (M = 9.3 years; SD = 6.9 years) and 49% (*n* = 23) of the relationship break-ups were partner initiated. Participants also completed the PHQ-9 (Kroenke et al., 2001), a commonly used depression screening tool, and more than half of the men (*n* = 27; 57%) scored five or above, which indicated mild to severe depression over the past two weeks. Of these 27 participants, almost half (*n* = 12; 44%) indicated having suicidal thoughts in the past two weeks (please see Table 1. Participant demographics).

**Table 1.** Participant demographics (*n* = 47).

Age (years) (range 26–70; mean 41)	*N* (%)
20–29	7 (14.9)
30–39	15 (31.9)
40–49	17 (36.2)
50–59	4 (8.5)
60–69	3 (6.4)
70+	1 (2.1)
Gender	
Male	45 (95.7)
Gender queer/Gender non-binary	2 (2.1)
Sexuality	
Heterosexual	38 (80.9)
Gay	7 (14.9)
Bisexual	2 (4.3)
Education (highest level completed)	
Some or all high school	4 (8.5)
Some college	8 (17.0)
Diploma or certificate	11 (23.4)
Bachelor’s degree	17 (36.2)
Postgraduate degree	7 (14.9)
Marital status	
Single, previously separated or divorced	22 (46.8)
Partnered or married (previously dated/common-law/divorced)	14 (29.8)
Single, never married	11 (23.4)
Relationship duration (Range=4 months - 28 years; M = 9.3 years; SD = 6.9 years)	
<1 year	4 (8.5)
1–5	12 (25.5)
6–10	16 (34.0)
11–15	5 (10.6)
16–20	8 (17.0)
21–25	1 (2.1)
26–30	1 (2.1)
Relationship break-up initiated by	
Participant	21 (44.7)
Partner	23 (48.9)
Unclear/mutual	3 (6.4)
Current living arrangements	
With children	12 (26.0)
Lives alone	10 (21.3)
Partner/spouse	10 (21.3)
Other family member(s) (e.g., parents)	7 (14.9)
Partner/spouse and child (ren)	4 (8.5)
Roommates	4 (8.5)
Fathers	
Fathers	24 (51.1)
Who do you talk to about your relationship break-up? (Check all that apply)	
Friend(s)	39 (82.8)
Healthcare professional Psychologist, therapist, counsellor, psychiatrist, psychoanalyst, family doctor parenting coordinator, social worker	23 (48.9)
Partner/spouse/new partner	18 (38.3)
Family	12 (25.5)
Facilitated peer group	5 (10.6)
PHQ-9	
None-minimal (0–4)	20 (42.5)
Mild (5–9)	12 (25.5)
Moderate (10–14)	8 (17.0)
Moderately severe (15–19)	6 (12.8)
Severe (20–27)	1 (2.1)
Suicidal ideation (past 2-week)	
Yes	12 (25.5)
No	35 (74.5)

### Data Analysis

**Figure 1.**
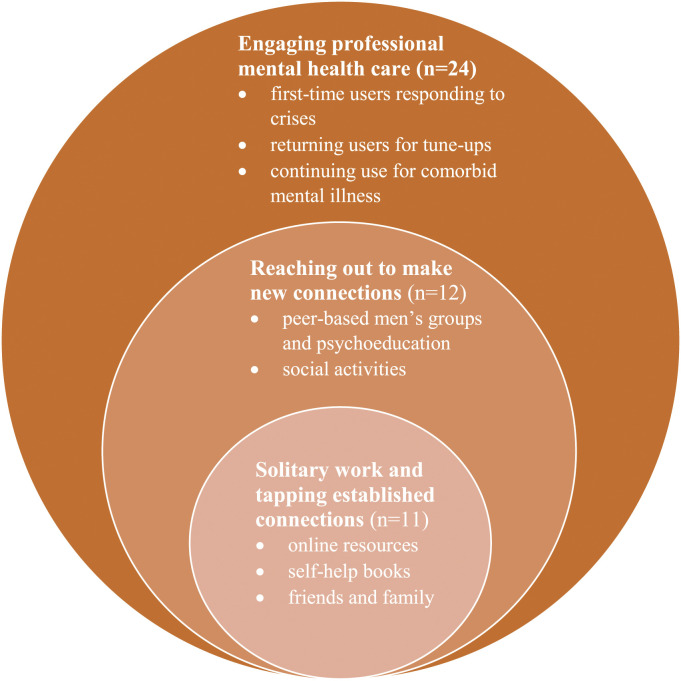
Mapping men’s help-seeking after a relationship break-up.

Each transcribed interview was read and summated to provide an overview including details about the relationship break-up and the men’s related help-seeking. In reviewing all the summaries and returning to the full interviews we began to address the research question, *What are the gendered dimensions of men*’*s mental health help-seeking after an intimate partner relationship break-up?* In further familiarizing ourselves with the interview data, discrete help-seeking patterns were noted based on the type of help that was accessed by the men. The initial coding schedule comprised two broad labels—self-help and professional help. These preliminary coded data were reviewed to develop a cohesive picture of informal and formal professional help accessed by each participant. Based on these assessments, three types of help-seeking categories were identified and defined: in-house, new objectivity, and self-triaging professional care services. Every interview was allocated to one category based on the participants’ help-seeking behavior. As we further defined the categories and analyzed the assigned interviews, labels and preliminary findings were developed for each emergent theme; (1) *Solitary work and tapping established connections* (*n* = 11 participants), (2) *Reaching out to make new connections* (*n* = 12 participants), and (3) *Engaging professional mental health care* (*n* = 24). Using a constant comparative approach (Charmaz, 2006), the interviews allocated to each theme were compared and analyzed to distil the patterns and account for variations within and across the three themes. Figure 1, mapping men’s help-seeking after a relationship break-up, highlights the connectedness between the themes depicting the men’s help-seeking. Participants were assigned pseudonyms; age and relationship details accompany illustrative quotes to orientate readers to the speakers.

## Findings

### Solitary work and Tapping Established Connections

Eleven participants limited their help-seeking to *solitary work and tapping established connections* following the break-up of their relationship. These participants ranged in age from 26 years to 57 years (M = 37.3; SD = 9.7), most had never been married (*n* = 7) and narrated relationships ranging from 4-month to 10-year (M = 5 years; however, the majority were <3 years; SD = 3.3 years). Solitary work included men busying and distancing themselves from painful or negative feelings about the break-up, and such efforts were routinely coupled with discreetly gathering resources to help move on from the relationship break-up. Chad, a 30-year-old man, spoke about laboring to accept his partner’s decision to end their 4-month relationship. Acceding his ex-partner’s realization that she had too quickly entered into their partnership following her previous relationship, Chad talked about the value of *doing* rather than *thinking* too much:

“I've been trying to focus on new hobbies a lot and doing things, like focusing on my work and I’m taking an insurance licensing course right now that’s keeping me busy. I’ve always felt like the busier I can get myself, the happier I am at the end of the day, and if I’m just sitting doing nothing that really wears on me and I end up just thinking about things and I don’t want to be thinking about things like that all the time because it just ends up making me sad.”

Chad was determined to distract himself from the sadness that flowed from the break-up. Striving to make his unexpected single life purposeful, he drew benefits from solitary recreation and career-related activities. As Harding and Fox (2015) suggest, like many men, Chad was likely self-protective in his avoidance of overthinking or feeling too much.

Solitary work also comprised efforts for understanding the demise of the relationship, making sense of feelings about the break-up, and, in many instances, scouring the internet for self-help resources. Bryce, a 33-year-old man, explained that he ended an 8-month relationship after he “came to know certain things” about his partner (the specificities of which he was careful not to disclose in the interview). In the weeks following the break-up, he searched the internet in hopes of better understanding his ex-partner and himself:

“I think reading about personality types, reading about ways people do certain things…So I think there’s so much online…But I think that’s something that’s worked for me really well…And following certain things on Instagram, I think it’s weird but it helps. I think just reading certain things and it’s like, ‘man, this makes so much sense.’”

Bryce’s solitary work focused on reconciling the fact he had been deceived by his ex-partner, affirming his swift action to end the relationship, and soothing the disproportionate hurt he felt in having severed a relatively brief (though promising) partnership. That Bryce did not disclose the details of his ex-partner’s transgression reflected the in-progress nature of respectfully interpreting, learning, and moving on from what had occurred.

Although solitary work could be therapeutic, being alone and independent could also gateway to risky practices including substance use. Brent, a 35-year-old-man, recalled using a “herculean amount of marijuana,” suggesting that living alone and the timing of his break-up during COVID-19 restrictions had fueled his overuse. Bryce similarly indicated that the pandemic induced boredom, and his struggle to accept the circumstances of the break-up, drove his substance use to the extent that it prompted him to reach out to friends:

“I was drinking a few more than I usually do which I’ve never done in the past…like a few nights, it was like, I think I’m going to have another beer and not because I needed to kind of forget about it, but it was just like I have nothing else to do. You’re kind of stuck at home. You can’t really socialize the way you typically do. So I think finding different ways to sort of accept it was what I was doing but I did reach out to my close friends…people that are in my network because I think at times you just need to.”

Brent and Bryce framed their solitary substance use as normative, qualifying that this was a short-term benign practice. However, as Turner et al. (2018) suggest, substance use, while normed as manly, can be (or become) self-medicating and injurious. Indeed, though Bryce rationalized his drinking, he decided to cut back and connect with friends as a means for better coping with the break-up.

In addition to solitary work, many men tapped established connections including family, friends, and social networks. In these instances, participants actively sought out and engaged others to talk about what had happened, and articulate what they were feeling about the break-up. Ryan, a 31-year-old man, detailed some turbulence in his relationship prompting him to leave his partner and their shared apartment. He subsequently reached out to a co-worker and suggested the “judgement free” and “supportive” conversations were especially helpful and calming. Chad, whose partner initiated the break-up, agonized about her change of heart. In response to his willingness to reach out and be vulnerable, Chad’s friends listened and empathized in reassuring him that he:

“was not this person who is incapable of happiness and…that there are good qualities about me.

In tapping established connections, Ryan and Chad valued being heard and reassured without the pressure to rationalize what was felt, or remedy issues underpinning the break-up. Key also, as Bryce suggested, was the need to have “*those conversations with them [friends] without completely dissing the other person [ex-partner].*” Herein maintaining respect for ex-partners was crucial, as were friends’ authentic efforts to listen without judging, criticizing (former partners), or pseudo-mediating past relationship conflicts or omnipresent grievances.

For some men, there were concerns that disclosing vulnerability and connecting to talk about the break-up could be traumatic in and of itself. Andrew, a 33-year-old father, explained his initial reticence and fears for debriefing with friends or family about his decision to leave his partner and their 3-year-old son:

“I knew that that was not the kind of relationship I wanted to be in, so I feel like, I thought that if I talked about it with friends, family, stuff like that, I’d get some pretty hard judgements back or whatever. Like ‘keep your house in order.’ The old ‘man up’ sort of garbage. That’s what I thought I would get, just super judged. I had this thing for the longest time of just fear of being judged, even though it really doesn’t matter. These days I know that. Back then, I really didn’t know that, so yeah there was definitely no talking about that with anybody, so that was all bottled in for sure.”

Andrew referenced a shift in his read of, and alignment to masculine ideals prescribing stoicism and self-reliance wherein he eventually reached out to family. Although shame and concerns for being judged a lesser man (and father) for leaving his partner and son initially silenced Andrew, he explained:

“my family was super supportive, super, super, super supportive. My sister was talking to me daily, she called me daily.”

Chandler (2021) suggests men’s talk can be muted by dominant masculine discourses that idealize men as stoic and unemotional, and for Andrew, the shame, grief and guilt flowing from the break-up slowed (but did not ultimately stop) him tapping established connections.

These participants were also reticent to extend their help-seeking to new connections or professional mental health care services. Some men were uncertain if they could engage with and/or navigate those care options. Brent, a 35-year-old man, who ended a 6-year long-distance relationship, explained:

“I think what was most value to me was one on one conversations with people whose opinions I trusted. I did them all face to face…I don’t think any group thing would have helped in that situation. I think I probably would have just clammed up…Like honestly since the pandemic hit I have thought I should probably go to therapy. That sort of thing seems quite expensive and I’ve heard a lot of horror stories and I’m not super sure how to navigate the healthcare system in order to make that work, if I just call up offices? so argh.”

Brent appraised the help of friends as sufficient in listing an array of reasons why he had not accessed other help. Though visibly emotional when disclosing that a lack of progression in the relationship toward having a child was the lynchpin for the break-up, when asked, Brent described how he had coped in the 6-month since the split:

“Time, honestly. Basically. That was kind of a matter of time and then having other things to distract myself with while I figured that out and processed it.”

In summary, men depending on *solitary work and tapping established connections* highlighted benefits and risks for containing their help-seeking with an emphasis on moving past the relationship break-up. The benefits of self-sufficiency and established supportive connections were tempered by the potential for maladaptive behaviors, and the isolating effects of concealing or downplaying distress.

### Reaching out to Make New Connections

Twelve participants, in addition to engaging various aspects of *Solitary work and tapping established connections,* extended their help-seeking by *Reaching out to make new connections*. These participants ranged in age from 28 to 52-year-old (M = 39.8 years; SD = 9.7 years), their break-ups involved relationships lasting 2–16 years (M = 7.2, most often >5 years; SD = 4.3 years), and most were currently single (*n* = 8). Men purposefully connected with individuals and/or groups they had not known prior to the break-up. For the most part, participants were invested in better understanding the demise of the relationship and their role in the failure of the partnership. Some participants accessed other men online who had experienced relationship break-ups seeking operational help (i.e., legal, financial settlement, and child custody) and strategies for recovering emotionally. Samuel, a 42-year-old father, explained:

“Talking to a stranger is probably better rather than talking to your family, because your family they can be there for you, but talking to a stranger, you get that different view, that mutual view of what should happen…I have reached out to a gentleman. He’s been through separation as well and he’s helping other men deal with separation. I’ve met with him once. I did a Zoom call with him last week and he helps men thrive after separation and that’s something that I want to do. I want to thrive. I want to be better. I want to be happier, but I have to have a plan in place to get there.”

Samuel signaled how impartial practical advice assisted him to anticipate and navigate what lay ahead (i.e., financial settlement and child custody orders) in purposefully mapping strategies to ensure his well-being during and after those processes. Samuel valued the man’s firsthand insights, and his willingness to help him through the break-up. As Addis and Mahalik (2003) suggest, men with direct experiences helping other men transition can norm help-seeking, and Samuel deeply appreciated being forewarned, and by extension better prepared to strategize coping with the break-up.

Participants also sought help from community-based groups for general and specific supports related to the break-up. Dylan, a 49-year-old father, in the aftermath of his second divorce, explained that an advertisement for an in-person group assertiveness course which provocatively asked “are you feeling depressed?” prompted him to enroll:

“I think it was four two-hour sessions after work…like adult learning…it was a very basic, low budget course…I just decided I’d do a short course to help myself…you know, even my most insightful friends don’t really know the true meaning of assertiveness and they don’t know how that kind of thing can help you. That really, that was the most positive thing I did, I reckon that I still use those techniques and think ‘how can I get the best outcome for everyone, not myself and not them’, I think like that - reflexively.”

Dylan foregrounded the limits his friends’ help in rationalizing his need to look externally to build his communication skills, and more specifically to negotiate the child custody arrangements accompanying his break-up. These efforts were prompted by deficits that Dylan recognized in himself as prolonging (and likely increasing) the relationship distress throughout and long after the break-up. He suggested that his wellbeing and the health of his ex-partner and children were aided by his assertiveness work, appraising the help as asset-building to better deal with his circumstances. Akin to Fletcher and St George’s (2010) assertion that men’s mental health help-seeking can comprise general self-help work, Dylan spoke to the usefulness of what he learnt, and how it had changed his mindset and demeanor for the better. It was also clear that Dylan was still working to recover from the break-up, and this included taking the time to re-establish his identity and independence:

“I guess I would never say never [to another relationship] but I mean…it’s like trying out for a football team and your head gets pushed down into the paddock and you get up, you have another go and you get slammed into the ground again. And then that keeps going on so why bother? I would think ‘what are the chances that I would find someone that likes…pottery, likes metal detecting, all the things that I do? - not very much.”

Many participants, including Dylan, were single, and reaching out to make new connections tended to focus on their recovery rather than searching for a new partner. Indeed, Dylan’s sports metaphor signaled his injury as current in dismissing the potential for another partnership. Set and steadfast in his interests, Dylan’s assertion about choosing to be single might also suggest some self-protection, especially given his involvement with a newly formed group of separated fathers who regularly shared the afore-listed activities (pottery and metal detecting) when they meet-up.

Some participants attended facilitated men’s groups that focused on shared support for men experiencing a relationship break-up. Jack, a 39-year-old father who was in the midst of family law legal proceedings when we spoke with him, explained:

“You know, when you turn up and your son says, ‘dad, why don’t you live here anymore?’ And it’s like, well, ‘how many times can my heart break?’ … I had to find ways to remind myself that it was the right thing to do [separate] and things like the men’s group were instrumental in that, having the opportunity to voice how I was feeling, how I was thinking, but then to hear from other men … and to know that the way I was feeling wasn’t some unique feeling, that it was quite common and that it was okay to feel that way.”

Jack’s guilt, self-doubt, and vulnerabilities beset the interview, and he suggested that the men’s group normed those emotions, and provided permission to express what was felt as well as witness similar self-disclosures by other men. The men’s group purposefully refrained from providing advice or espousing quick fixes, though Jack suggested the meetings had enabled him to reconcile the dispute between his heart and head to sustain his decision to leave the relationship. The group format normed men’s feelings and need to express what they felt in deciding (and rationalizing) their actions. Jack continued to defend the men’s group work as an important subaltern masculinity that was rich in therapeutic value:

“Just because you go and talk to other men about how you’re feeling, doesn’t make you any less of a man in the stereotype way in there. It’s just farcical that there’s so many people who seem to think they have to sort it out themselves and often you don’t have to sort anything out, just acknowledging your feelings, acknowledging how you’re feeling at the time can be more powerful than sitting there trying to fix whatever you think is broken because it might not actually be broken. The fact that you’re feeling it could mean that it’s working fine.”

Jack contested masculine norms that idealize men as unemotional in asserting that *not feeling* was the primary issue (and injury) for most men. Jack’s counter-narrative for doubters argued against the social policing of what men can and do feel and disclose; his assertions positioned the group’s masculine milieu as strength-based and purposeful in providing restorative mutual help.

Although reaching out to make new connections provided benefits, participants stopped short of sustaining professional mental health care. This reflected the self-assessed absence of severe mental illness symptoms, as well as many men’s preferences for self-selecting alternative strategies for moving forward. Mick, a 46-year-old father, who had previously experienced suicidality contrasted the problem-based approach of professional counseling with his men’s group self-development work, in asserting that his needs were best met by the latter:

“Therapy sometimes can backfire on you in these situations [separation] because the therapist constantly highlights the problem for an hour…they just talk constantly, talk about this problem, like, you know, relationship breakdown for a one-hour session whereas all these personal development things, they are more working on you instead of the problem…the fix is you instead of the problem.”

Mick’s experience resonates with Seidler & colleagues’ (2021) assertion that ill-fitting professional mental health care services can lead men to dropout of treatment. That said, Mick did not abandon his search for help; rather his focus on self-work led him to a facilitated men’s group.

In summary, *Reaching out to make new connections* revealed participants’ preference for practical, objective information and self-help that solitary efforts and established connections were unlikely to provide. The help solicited also extended beyond empathetic listening to navigate the complexities (i.e., legal, social, emotional, and financial) inherent to the break-up of longer-term relationships and men’s interests in self-improvement.

### Engaging Professional Mental Health Care

In addition to accessing elements of the help detailed in the *Reaching out to make new connections* and *Solitary work and tapping established connections* themes, 24 men engaged professional mental health care throughout their relationship break-up. These participants ranged in age from 26 years to 61 years (M = 44: SD = 10.3) and half (*n* = 12) had been involved in relationships or marriages lasting 9 years or longer (Range=9 months-28 years; M = 9.89 years; SD = 6.7). Sixteen of these men were fathers sharing custody of their children with an ex-partner. Five men were currently living with a new romantic partner and 19 reported living alone, with roommates, or a family member. The participants’ pathways to, and length of engagement with, professional mental health care services varied—though their help-seeking intent was consistent—to effectively cope with the break-up, procure skills for self-managing their mental health, and/or garner self-growth to improve future relationship outcomes. Some men accessed services for the first time after the break-up, whilst most participants re-engaged or continued their use of professional mental health care to address challenges specifically related to the partnership ending.

Five men accessed professional mental health care for the first time after realizing they needed expert help for dealing with the break-up. Ian, a 44-year**-**old father, whose 18-year marriage ended with his wife’s infidelity, stressed the importance of getting professional mental health help, in addition to making lifestyle modifications to cope with the relationship ending:

“If they can obviously do stuff to maintain their mental health it’s like, you know, talking to friends and exercising, and delete Facebook and hit the gym but they also need to have professional therapy as well because like, there’s a reason why you go to a psychologist because they’re trained to do it, and they give you the skills that you need to push through it.”

Ian explained that therapy helped him shift his focus from the tension filled relationship with his now ex-wife to center on the wellbeing of their children. Working with that base, Ian detailed how he was expertly coached by his psychologist to transition out of the marriage while retaining some sense of agency in negotiating the co-parenting arrangements. Ian also clarified that he “did not actually talk to my family about it [the infidelity] because I…did not want my family judging her [ex-partner].” As Seidler et al., (2020) suggests, belief in professional help is key, as illustrated by Ian’s appraisal:

“I think engaging in quality therapy is essential. I mean because unless you have got some skills to change your mindset things aren’t goanna go well at all.”

Chuck, a 33-year-old, lamented knowing his relationship was anguished well before it ended with the news that his partner of 10-years had had an affair with a co-worker. Explaining that the trauma of the split prompted him to seek professional mental health care for the first time, Chuck rationalized his delayed help-seeking as the by-product of being unable to articulate the problem [s] fueling the distress in his relationship:

“I didn’t know what the real problem was, so to seek help you have to know what you’re trying to fix, right. I kind of learned that after the relationship breakdown, I hit my lowest point and then I just started seeking…I was pretty much dedicating 24 hours a day, literally because I wasn’t sleeping, to therapists, reading materials, group therapy, divorce coaching…. I learned a lot during that process about how to look for help.”

Chuck’s break-up induced anxiety and depressive symptoms rallied his search for professional help whereas the distress characterizing the relationship was normed. Such crisis driven help-seeking echoes Struszczyk and colleagues’ (2019) findings that physical and/or mental pain is often known and monitored by men but not addressed until it is severe and debilitating.

Based on previous positive experiences with professional mental health care, seven participants re-engaged services after their relationship break-up. Paul, a 28-year-old man, whose partner of 5-year ended their relationship when they were on the verge of moving in together, worked with a psychologist for 6-month after the break-up. Norming his use of professional help, Paul referenced previous therapy after his parents divorced, wryly declaring, “*I’ve had my fair share of psychiatrists and psychologists growing up.*” Paul also clarified that the absence of friends and family support amid the lingering weight of needing to accept the break-up motivated his return to professional help:

“It was a very elongated problem and wasn’t going away any time soon, I think there came a point where - I’d seen…one counselor, one psychologist - I decided that things were not looking so reachable round trying to get back with her [partner] – that [therapy] was more of a moving on process. So at that point trying to just kind of make it more about myself and grieve rather than deny.”

Paul highlighted “compassion” as the most important ingredient in professional help; he valued being heard and having someone to talk with in conceding the end of the relationship. Other participants craved practical strategies for coping with recurrent mental health issues emerging with the break-up. Russel, a 33-year-old father who had previously experienced anxiety and depression, re-engaged individual counselling during the custody disputes at the end of his 8-year marriage. He explained his preference for “*practical counselling, not just, you know, kind of whining counselling*” in detailing his use of cognitive behavior therapy (CBT):

“I found it [CBT] helpful in the past when I had issues in first year university. I still have those tools now, but I need a refresher. That one [CBT] I find helpful. Just action plans…these are the little baby steps I’m going to take. Just having certain things that you set for yourself, little things, not big things because I think it’s not easy to do big things when there is a huge thing already happening…So I’m hoping that’s going to get better as I keep transitioning.”

Russel planned to continue with individual counselling, contingent on being able to afford it, to regain his mental health, stay employed and effectively co-parent. For participants returning to professional help, a myriad of other mental health promotion strategies were integrated including self-help books previously reported by Doss et al. (2009), physical exercise and connecting with friends and family for support. Purposeful, the goal was to augment and leverage professional care to address the acute injury (and crisis) and return to self-managing their mental health.

Twelve men experienced mental health challenges prior to and during their relationship for which they continued to engage professional help after the break-up. Phil, a 35-year-old man, regularly saw a counsellor, and his partner’s preference for a polyamorous partnership emerged as an issue to address in therapy. Reflecting on his 3-year relationship, Phil explained that despite individual and couples’ counselling, their “pursuer and withdrawer dynamic” eventually ended the partnership:

“I had gone to see a counselor personally just to discuss stuff from work and to discuss little things that come up and obviously when you’re talking about your personal life, you’re going to talk about the person you’re in a relationship with. So she [partner] actually ended up coming to some of my sessions and we did kind of like a relationship counselling just to assess those things and it was helpful, but it still came back to this theme of her being kind of curious and feeling like a lack of intimacy from me and me truthfully pulling away as well…it [counselling] didn’t really change the fact that there was still some sexual frustration there and tension that wasn’t being addressed.”

Phil’s post break-up counselling focused on deciphering his role in the mismatch, in particular his concurrent complicity and regret for participating in the polyamorous aspect of the relationship. Addressing his lack of authenticity in failing to express his desire for a monogamous relationship, professional help tooled Phil to address anxiety attachment issues deemed to have impeded all of his intimate relationships. For Phil, and many participants, continuing with professional mental health care services focused on self-work well beyond the break-up trauma.

Some men’s mental illnesses were especially complex. Travis, a 33-year-old man whose relationship ended after 3-year, had experienced anxiety and depression since childhood. Conceding his mental illness challenges contributed to the demise of the partnership, Travis explained that the break-up demanded intensive therapy to address the psychological impacts:

“It [counselling] definitely helped me get back on my feet, on a basic normal level, like so I can sort of function in a workplace environment properly again, and luckily I’m… working with people and getting out and about again; functioning like a normal adult again. There was a point where it seems like we were slowing down progress and I did go on medication, and that helped. Then I’ve had to do the whole medication dance where it’s like ‘hey this one doesn’t work, this one works’, I’ve done all that. It definitely helped me get better.”

Travis accepted the need for ongoing professional mental health care. Indeed, his help-seeking was finely tuned to respond to changes including the pain of the break-up, which recursively interacted with his longstanding anxiety and depression.

Men *engaging professional mental health care* revealed diverse usage patterns, and complex and contextual help-seeking patterns. Although accessing professional help affirmed the significant injury potential of a relationship break-up and men’s crisis management, it was also a staple for many men who experienced ongoing mental health challenges. Herein, a relationship break-up was but one intersecting (albeit pivotal) issue norming many men’s re-engagement or ongoing need for professional mental health care.

## Discussion and Conclusion

The current study findings highlight the connections between masculinities and men’s mental health help-seeking—the sum and parts of which affirm longstanding evidence about the injurious potential of intimate partner relationship break-ups (Evans et al., 2016; Kposowa, 2003; Shiner et al., 2009; Stack & Scourfield, 2015). Curiously, despite dire downstream outcomes including severe mental illness and suicide, amid perennially newsworthy reports of murder-suicides that are linked to disrupted relationships (Kalish & Kimmel, 2010), there has been little attention to men’s mental health help-seeking (or needs) in the specific context of relationship break-ups. The findings drawn from the current study address these knowledge gaps, albeit by calling into question decades of research that has positioned professional care as the only help that counts, along with claims that men who align to specific masculine norms are likely universally deficient in their mental health help-seeking. In what follows we draw on the study findings to discuss three key points.

First, it is fair to say that disrupted intimate partner relationships, irrespective of the duration and complexities of the partnership, prompted men’s pursuit of restorative mental and emotional health. Moreover, men’s agency in trialing and determining *their* help sources, might be reasonably argued as reflecting strength-based, asset-building masculinities. Although conceding the risks inherent to some men’s solitary self-health practices (i.e., substance use, concealment, and downplaying of emotional pain) and the potential for underestimating the need for professional help, for the most part, the findings run counter to deficit models that isolate self-reliance as *the* explanatory masculine norm for men’s resistance to seeking mental health help (Mahalik & Di Bianca, 2021; Mahalik et al., 2007; Pirkis et al., 2017). Rather, affirming previous qualitative work (Emslie et al., 2006; Ferlatte et al., 2019; Krumm et al., 2017; Oliffe et al., 2021; Player et al., 2015; Staiger et al., 2020), our findings highlight the potential for men to be self-directed and agentic in their pursuit of, and openness to receiving specific and diverse forms of help. Indeed, participants searched and mobilized solitary (self-help books and the internet), existing (family and friends) and new psychosocial resources (men’s groups and education classes) amid self-triaging professional help as short and ongoing projects. With regard to self-help books, several titles reported as influential by Doss et al. (2009), John Gray’s (1992) *Men are from Mars and Women are from Venus* and Gary Chapman’s (2004), *Five Love Languages*, featured in the current study findings*.* We agree with Doss et al. (2009) that even though some popular titles have the potential to entrench limited perspectives on gender and gender relations, select titles can provide men with opportunities to build their relationship skills.

The contrast between quantitative and qualitative representations of masculine self-reliance in men’s mental health help-seeking may reflect differences between reductionist interpretations of responses to pre-determined survey items and the inductively derived meanings drawn from freeform interview narratives. That said, it may also be an unhelpful overreach to suggest men’s responses to general help-seeking survey items (e.g., “I never ask for help” and “It bothers me when I have to ask for help”; Pirkis et al., 2017) tally to reveal their conformity to masculine self-reliance—let alone predict universal deficits in some men’s mental health help-seeking. In addition, while attribution for men’s mental health help-seeking is consistency assigned to the influence of partners (Salvatore et al., 2020), our findings reveal how, in the absence of a partner, men can (and do) creatively find informal help and/or formal professional help. Most often with the goal of bridging to self-management, participants’ help-seeking indicates masculine self-reliance can work for men in the aftermath of a relationship break-up.

Second, in line with Fletcher and St George (2010), we argue for an adaptive explanation that disbands with professional mental health care as the only form of legitimate help—and by extension the measure by which men’s help-seeking is evaluated. Participants in the current study rallied (and relied on) emotional support from friends and family, purposefully extended new psychosocial resources for objective help, and/or engaged professional care. Men’s widespread use of self-help relationship books affirms previous findings (Doss et al., 2009) and their uptake of peer and community-based group programs, e-relationship, and mental health resources (Entwistle et al., 2021; Ogrodniczuk et al., 2021; Oliffe et al., 2020) underscores the expansive nature of men’s help beyond the predominance of professional mental health care services. Pre-COVID-19 work by Best and colleagues (2016) indicated that young men’s mental health help-seeking relied on connecting with trusted friends online and offline, and these pathways facilitated emotional disclosure, while formal online help was accessed and deeply valued for its anonymity, confidentiality and content. These trends, perhaps especially e-resources and connectedness, are likely to grow in influence and popularity with COVID-19 restrictions, and all that follows the pandemic. Men’s shifting mental health help-seeking also reminds us of the need to disband with framing men as passive recipients of gender socialization and masculine ideals. Instead, there is a need to recognize (and affirm) men as active agents intent on managing relationship break-up challenges with “options constrained by real-life social arrangements” (Fletcher & St George, 2010, p. 50).

Third, our findings confirm complexities in men’s estrangement and engagement with professional mental health care services. Resonating with Seidler et al., (2018, 2020, 2021), men’s trust, expectations, and perceived benefits were lynchpins to the normative framing for engaging professional help. Adding to this, participants’ self-triage revealed the possibilities for first-time, returning and continued use of professional mental health care to trouble the binary of being in or out of treatment. To address the risks inherent to accessing professional care for crisis-management, there is no doubt value in lobbying bi-annual check-ins as preventative and maintenance measures. These connections can be bridged to men’s self-management preferences with an ever-ready option to more fully and frequently engage professional help.

In terms of limitations, the cross-sectional study design and absence of partner and/or help source data does not reveal what might change over time in regards to men’s mental health help-seeking, amid the confines of relying on men’s self-report in what is clearly a gender relations issue (both in terms of the relationship break-up and men’s help-seeking experiences). Solely relying on online recruitment may have influenced the sample, and by extension the specific findings drawn from that participant subgroup. Nonetheless, the current study findings provide a foundation to build longitudinal studies inclusive of triangulated data sources to further advance the field.

In conclusion, though the current findings demonstrate that men experiencing relationship break-ups derive considerable benefit from sourcing informal and formal help, mental illness remains a significant health concern for men exiting intimate partnerships. Recognizing distressed and disrupted partnerships as key insertion points for men’s mental health promotion programs is critical. Upstream, as Spencer and Anderson (2021) suggest, there are protective benefits in equipping men with relationships skills through online programs. Herewith, we might reduce the potential for dire mental illness outcomes through prevention as well as anticipating and supporting men through the mental health challenges that are known to accompany relationship break-ups.
